# Altered mental status, an unusual manifestation of early disseminated Lyme disease: A case report

**DOI:** 10.1186/1752-1947-1-62

**Published:** 2007-08-09

**Authors:** Shiven B Chabria, Jock Lawrason

**Affiliations:** 1 Department of Medicine, Waterbury Hospital, Waterbury, Connecticut, USA

## Abstract

Early disseminated Lyme disease can have a myriad of central nervous system manifestations. These run the gamut from meningitis to radiculopathy and cranial neuropathy. Here we present a case that manifested with only acute mental status change in the setting of central nervous system involvement with Lyme disease. A paucity of other central nervous system manifestations is rare, especially with positive serum and cerebrospinal fluid markers. This article underscores the importance of a high index of clinical suspicion in detection of Lyme disease related manifestations in endemic areas.

## Background

Lyme disease is a multisystem inflammatory disease caused by spirochetes, known collectively as Borrelia burgdorferi, which are spread by the bite of infected Ixodes ticks. Lyme disease was first described in studies of an outbreak of "juvenile rheumatoid arthritis" in Connecticut [1]. It is endemic in the states of Massachusetts, Connecticut, Maine, New Hampshire, Rhode Island, New York, New Jersey, Pennsylvania, Delaware, Maryland, Michigan, and Wisconsin [2]. The clinical manifestations are usually categorized into early localized, early disseminated, and late. Early disseminated disease occurs days to months after the tick bite and may be the first manifestation of B. burgdorferi infection, without preceding erythema migrans. Alternatively, there may have been antecedent erythema migrans and/or systemic complaints in the early localized phase. Early disseminated Lyme disease can have many central nervous system manifestations. These run the gamut from meningitis to radiculopathy and cranial neuropathy [3]. The range of sequelae in untreated early disseminated Lyme disease include rheumatologic phenomena (monoarticular or oligoarticular arthritis) in 60 percent neurologic manifestations (usually facial nerve palsy) in 10 percent and cardiac complications (atrioventricular block) in 5 percent [4]. The presence of altered mental status as the sole and only manifestation of central nervous system involvement in early disseminated Lyme disease is presented here for discussion.

## Case presentation

An eighty four year old man presented to the emergency room after his wife found him to be behaving differently than usual. He carried diagnoses of hypertension, history of stroke and mild to moderate dementia. The wife noted him to be hallucinating three to four days prior to presentation. Oral intake had diminished considerably and he was found to have decrease in functional capacity. There was no history of fevers, chills, rigors, nausea or vomiting. The patient and his wife had returned from Maine approximately 4 weeks ago where they had been vacationing. There was no recent or remote history of travel outside the country. No changes were made recently to his medications.

Physical examination revealed a pleasant elderly gentleman. He was hearing impaired at baseline. His vital signs including oxygen saturation were within normal limits. Patient was noted to be agitated, confused and at times mumbling incoherently. The wife noted this was different from his normal baseline which was forgetful but coherent. No icterus was noted though there was minimal conjunctival pallor. Respiratory exam and cardiovascular exam was unremarkable. No focal cranial nerve deficits were noted and there was no neck stiffness nor was any nuchal rigidity appreciable. Both Kernig's and Brudzinski's sign were negative. The patient would mumble incoherently which his wife noted was new since he could talk at baseline. Tongue was midline. Examination of his extremities showed a bruise like lesion on the antecubital area of his left arm.

Laboratory exam on presentation showed anemia with a hemoglobin of 10.7 g/dl thrombocytopenia with a platelet count of 144 thousand/mm^3 ^urine analysis was completely normal. The patient did have hyponatremia at 127 mmol/L with hypochloremia of 91 mmol/L and the serum was hypo osmolar at 267 mOsm/k. A CT scan of the head done on presentation showed chronic white matter changes without evidence of infarcts, tumors or organic brain lesions. RPR was negative, TSH was normal, B12 and folate levels were within normal limits.

Through the course of the next twenty four hours the patient was unchanged. He at no point showed signs of infection, the WBC count and temperature remained within normal limits. The hyponatremia corrected on hydration. The patient was seen by the neurologist who recommended an EEG and MRI be done. The EEG showed diffuse slowing consistent with encephalopathy and the MRI showed old infarcts in the left fronto-temporal lobes. Upon further questioning the wife regarding the bruise on the patients left arm the wife mentioned that this probably was a "black fly" bite that he had sustained during his trip to Maine about a month ago. However nobody had actually seen the fly or other insect bite the patient. They hadn't sought treatment for it since it seemed to be improving without intervention. She was unable to describe the initial rash fully but didn't note a central clearing or a bull's eye configuration to it.

Based on clinical suspicion this gentleman underwent a lumbar puncture, and both peripheral blood samples and cerebrospinal fluid samples were sent for Lyme western blot. CSF chemistries were remarkable for an elevated protein level of 101 mg/dl with normal glucose. Cell count showed a WBC count of 43/mm^3 ^with 83% mono nuclear cells. Red blood cells were absent. Based on this the patient was empirically started on Ceftriaxone two grams once a day intravenously.

Over the next few days the patient's mental status was noted to improve. He was more coherent and awake much to the delight of his wife. The CSF was negative for VDRL and Herpes simplex. Both CSF and peripheral blood ELISA with reflex Western Blot tests were positive. Lyme IgG via Western blot was negative. However, Lyme IgM via Western Blot was positive for IgM antibodies against Borrelia burgdorferi antigen 23 and 41. Based on current guidelines for interpretation of serologic tests in Lyme disease this was viewed as a positive serologic diagnosis [5]. The patient improved considerably over the next few days and was discharged to an extended care facility to complete a four week course of antibiotics.

## Discussion

This case illustrates the importance of an index of suspicion for the diagnosis of Lyme involvement of the central nervous system in a patient who at baseline had dementia. The authors live and practice in Connecticut which is a Lyme endemic state; as is Maine. The absence of other attributable causes and the presence of an atypical rash which looked more like a bruise prompted the authors to look for possible central nervous system involvement in Lyme disease. The initial CSF examination looked suspicious for aseptic meningitis or Lyme disease. The choice to treat awaiting serologic confirmation was based on data suggesting that delay in treatment might lead to irreversible neurological sequelae. Also if the clinical picture is anything but classical neuroborrelosis a lumbar puncture with appropriate serological testing should precede treatment of Lyme disease [6]. This patient improved over the course of his inpatient stay. In general a lack of clinical response should prompt investigation into an alternate etiology [7]. Isolated facial nerve palsies can be treated with oral regimens of amoxicillin, doxycyline or cefuroxime. But involvement of the meninges and or of the cerebral parenchyma require an intravenous third generation cephalosporin. A randomized study from Sweden suggests that oral doxycycline and intravenous penicillin may be equally effective in the treatment of CNS Lyme disease since the patients in both groups did very well [8]. However, it is difficult to extrapolate from a European experience to the United States given differences in strain of the organism, and predominant immunogenetic types of the patients at risk. Three to four weeks of antibiotics suffice in most cases.

## Conclusion

This case represents the complexity of medical decision making in cases where few physical clues could be relied upon. In the absence of typical physical and historical findings the rash which was not typical of erythema migrans led to the postulation of possible Lyme disease. The endemic nature of Lyme disease in the region coupled with an atypical rash finally led to this abstruse diagnosis.

## Competing interests

The author(s) declare that they have no competing interests.

## Authors' contributions

SBC was involved in the treatment and management of this case. JL was involved in the review and preparation of the manuscript and provided sub specialty advice and opinion. Both authors read and approved the final manuscript.
